# Surgical Clinic

**Published:** 1891-02

**Authors:** J. H. Edgington

**Affiliations:** First Clinical Assistant


For Daniel's Texas Medical Journal.

### SURGICAL CLINIC—MEMPHIS HOSPITAL MEDICAL COLLEGE.

### Excision of Sup. Maxilla — Herniotomy — Perineal Section for Urethral Stricture.

CLINIC OF W. B. ROGERS, M. D., PROFESSOR OF SURGERY.

[Reported by J. H. Edgington, First Clinical Assistant.]

CASE I. Fibroid tumor of left sup. maxilla; excision. Patient, Hudson B., 14 years, male, black; presented a tumor of five years existence, but which had attained the greater part of its size within the past two years. The growth involved the left upper jaw, presenting a very great deformity, protruding at least three inches forward, while superiorly it pressed visibly upon the orbital fossa. The left nasal cavity was completely occluded and the oral cavity was so encroached on that the mastication of solid food was next to impossible. Several of the cuspids were scattered along the lower border of the tumor but all outline of the alveola had disappeared. It was noted that the canine tooth of that side was wanting, but the parent explained that it had been extracted by the family physician. There was no ulcerated or inflamed patch visible. No pain was complained of, nor was there any tenderness detected, even on very firm pressure, made at numerous points. To the touch the tumor was extremely hard, impressing the idea that it was due to hypertrophy of the osseous tissue, probably brought about by an inverted tooth. There was no sign of a cyst in connection with the growth, hence it was pronounced a solid tumor, either osseous or fibrous in structure, probably the former. There was no glandular involvement nor other signs of malignity; the patient's general health good, hence excision of the mass was pronounced the proper operation.

*Operation:* Patient fully anæsthetized. Langenbeck's incision was made and flap turned back. Free hemorrhage necessitated the liberal use of hemostatic forceps. The bone forceps were then applied at floor of left nostril, cutting roof of mouth, and near inner angle of orbit but below the floor of that cavity, cutting the nasal process of sup. maxilla. At this juncture there was found a small crevice between the growth and malar bone, into which crevice a bone elevator was pressed and the tumor pried out, leaving behind the smooth surface of posterior wall of antrum, which limited the tumor, though pressed back well into pterygoid region. The floor of orbit was not removed, but the outer wall of nasal cavity had completely disappeared, leaving only the Schniderian membrane flapping in and out at each respiration. There was literally no blood supply to the tumor proper, which appeared to be a genuine simple fibroma, which had originated in the antrum and by pressure expanded that cavity and caused absorption of its walls. The cavity was packed with gauze and the flap replaced and held in situ by silk-worm gut sutures.

Ten days later the patient was sitting up and rapidly convalescing.

CASE II. Stricture of urethra—internal and external urethrotomy.

Patient, Ody T., 34 years, was sent from Mississippi for treatment of stricture. Examination with urethrometer showed urethra with calibre equal No. 20 American. Three inches from meatus a stricture, calibre 10, while perineal urethra barely measured No. 6, American size, and was corded and hard to the touch externally. There were symptoms of an inflamed bladder; frequent desire to pass water, which contained some pus cells. There were no elements in the urine indicative of kidney implication. Boracic acid, five gr. every four hours, had been given for two days. Patient was anæsthetized, and Rogers' urethratome passed, bulb tormed, and during withdrawal stricture in perineal urethra incised. A staff was then passed to bladder, patient put in lithotomy position and perineal section of full length of membranous urethra made. The stricture was thus divided its full length and a No. 20 sound was passed from meatus to bladder. Patient sent to bed.

Three days later a No. 20 sound was introduced without any trouble and one week later patient was sitting up and doing nicely.

Case III. Inguinal hernia—sac filled with blood.

*Operation:*—Patient, Jake G., male, 37 years of age, general health always had been excellent. Had had well marked inguinal hernia of left side; the hernial mass had not extended into scrotum for years but had been fixed just in front of os pubis and irreducible for at least six years. Usually gave no trouble; did not attempt to wear any truss. Four weeks ago while lifting a firelog, felt a slight pain in hernia followed immediately by very great increase of size of hernia; apparently coming down and filling the scrotum to size of large cocoanut. No fever; no strangulation nor pain followed, nor was he very much inconvenienced by this sudden increase in size of hernia. Bowels moved regularly and he continued his daily work.

Examination showed a very hard tumor within the scrotum; irreducible, painless. The neck of the tumor was very dense; the body slightly elastic. There was flatness on percussion, the color of the skin—black—precluded translucency. It was argued that so sudden a formation of so large a hernia ought to have been followed by symptoms of strangulation. A hypodermic syringe was used to explore the elastic portion of the growth, and a syringe full of blood, dark, was drawn out. It was clearly a hemorrhage into the hernial sac.

An incision was made the full length of the tumor, and a pint of dark blood containing many clots poured out. The hernia proved omental, a large piece of which was impacted in the canal, where it had firmly grown, having doubtless been there for many years. It was explained that no better result in the way of a radical cure could be asked than the plugging by this large mass of omental tissue. The ends of the omental mass were ligatured and excised. A portion of the hernial sac was cut away; the remainder packed with gauze, wound dressed and patient sent to bed.

Two weeks later patient presented to class, wound healing nicely.

